# Effect of Chromostereoscopic Stimulus on Accommodative Response and Subjective Perception

**DOI:** 10.22599/bioj.515

**Published:** 2026-03-04

**Authors:** Vivek Suganthan Ramasubramanian, Kartheyaeni Jothi Madhavan, Shahid afridi Hyder Ali, Johan raj Jeyaraj, Swetha Selvakumar

**Affiliations:** 1 SRM Institute of Science and Technology, IN

**Keywords:** Display Mode, Chromostereopsis, Dark Background, Accommodative Response, Lag of Accommodation, Subjective Preference

## Abstract

**Purpose:**

The phenomenon of chromostereopsis, where colours are perceived at different depths due to the eye’s optics, creates a potential conflict during accommodation. This prospective, cross-sectional study aimed to investigate the effect of different chromostereoscopic stimuli on the objective accommodative response and subjective user comfort.

**Methods:**

Thirty young, healthy adults (mean age 19.83 ± 1.18 years) read text passages presented on an iPad at a 50 cm viewing distance. Stimuli included red, green, blue, yellow, and mixed-colour text on a black background, compared to a standard black-on-white baseline. Accommodative lag was measured objectively using an open-field autorefractor, while subjective ratings of perceived depth and screen readability were collected via questionnaire.

**Results:**

A significant main effect of stimulus colour on accommodative lag was found (p < .001). The short-wavelength (blue) stimulus induced the greatest mean accommodative lag (0.61 D). Conversely, the long-wavelength (red) stimulus produced the smallest lag (0.18 D), indicating the most accurate accommodative focus. Subjective data strongly corroborated these findings, with blue being perceived as most distant and most difficult to read (76.67% and 73.33% of participants, respectively), while red was perceived as closest and yellow was perceived as the easiest to read (53.33%).

**Conclusion:**

Colour is a critical factor in visual ergonomics for dark-themed interfaces. A pure blue stimulus on a black background acts as a poor driver for accommodation, leading to significant focusing errors and a diminished perceptual experience. These findings provide a physiological basis for user interface design guidelines, suggesting that the use of short-wavelength, saturated text for reading tasks should be avoided to optimize visual comfort and performance.

## Introduction

In our increasingly digital world, a significant portion of daily activity, from work and education to social interaction, is mediated through electronic displays (Malter and Rindfleisch, 2019). This has led to a significant increase in sustained near-work, bringing visual ergonomics to the forefront of workplace health and safety. This prolonged visual demand is directly linked to a cluster of symptoms, collectively known as digital eye strain or computer vision syndrome, which includes eyestrain, headaches, and blurred vision, ultimately impacting user comfort and productivity (Kahal *et al*., 2025). This sustained engagement with near-work tasks also places a constant demand on the visual system, particularly on the mechanism of accommodation (Wagner *et al*., 2019). The accuracy of this process is critical for visual comfort and performance. While various factors contribute to this near-work strain, a critical and often overlooked element is the ergonomic design of the user interface itself, particularly the choice of colour and polarity, which directly affects screen readability and the physiological load on the visual system (Dobres *et al*., 2017; Muhamad and Mokhtar, 2024).

**Figure 1:**
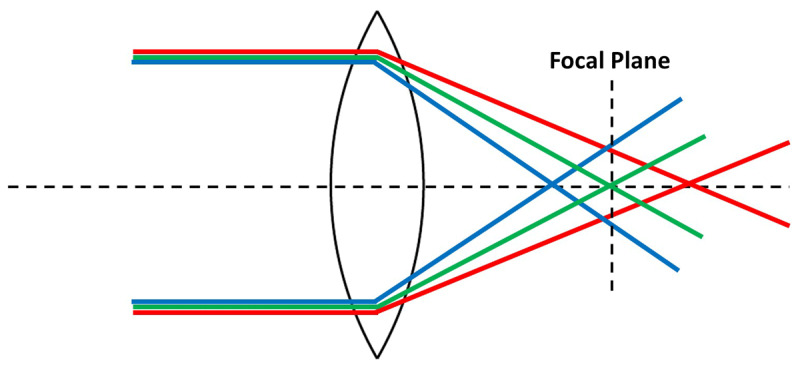
Schematic representation of LCA in the human eye. Short wavelengths (blue) are refracted more strongly and focus anterior to the retina, while long wavelengths (red) focus posterior to the retina. The interval between these focal points creates the chromostereoscopic depth cue. (LCA: Longitudinal Chromatic Aberration).

The challenge of maintaining clear focus on digital screens is further complicated by the optical properties of the human eye, particularly chromatic aberration. Chromatic aberration is the natural inability of a lens to focus all colours at the same point (Thibos *et al*., 1990). In the eye, this manifests primarily as Longitudinal Chromatic Aberration (LCA), where shorter wavelengths of light (e.g., blue) are refracted more strongly by the ocular media and come to a focus in front of longer wavelengths (e.g., red), as shown in Figure 1 (Howarth and Bradley, 1986). Quantitatively, this LCA amounts to approximately 2.00 D across the visible spectrum (400–700 nm) (Thibos *et al*., 1990). For the specific RGB wavelengths typically used in digital displays, this creates a dioptric interval of approximately 1.25 D to 1.50 D between the red and blue focal planes, creating a significant challenge for the accommodative system. This physical separation of focal points along the visual axis gives rise to a compelling perceptual effect (Karwoski and Lloyd, 1951). This phenomenon is known as chromostereopsis, which is defined as the visual illusion where colours presented on the same two-dimensional plane are perceived to lie at different depths (Allen and Rubin, 1981; Summersgill and Keating, 2011; Thompson *et al*., 1993; Westermann, 2022). Typically, due to LCA, red objects appear closer to the observer, while blue objects appear farther away against a black background and vice versa in a white background.

When viewing coloured text or graphics on a display, chromostereopsis can create a conflict for the accommodative system. The brain receives depth cues from colour that may contradict the physical distance of the screen, potentially influencing the accommodative response (Seidemann and Schaeffel, 2002; Thompson *et al*., 1993). When the eye’s accommodative response perfectly matches the stimulus distance, or accommodative demand, vision is sharp and effortless. However, a mismatch can occur, resulting in an accommodative lag (response is less than demand) or an accommodative lead (response is greater than demand), both of which can contribute to visual symptoms like blur, headaches, and eyestrain (Wajuihian and Hansraj, 2015; Zaman *et al*., 2025; Labhishetty *et al*., 2021).

Previous research has established that colour and contrast play a significant role in visual performance and comfort. For instance, Jiménez *et al*. (2020) found that blue-red colour combinations increased accommodative response, while other studies have investigated the effects of font size (Landrum, 2009; Zaman *et al*., 2025), contrast polarity (Molina *et al*., 2019; Muhamad *et al*., 2023), and display type (Hynes *et al*., 2022; Zhou *et al*., 2021) on accommodation and vergence. However, many of these studies did not systematically isolate the effects of specific chromostereoscopic stimuli—such as individual colours presented against a neutral, dark background—which are increasingly common in modern user interfaces (e.g., ‘dark mode’) (Damnjanović *et al*., 2024).

Despite the prevalence of dark mode, a significant gap exists in the literature regarding the precise nature and magnitude of the accommodative response to isolated chromostereoscopic stimuli. These designs frequently use coloured text that can trigger chromostereopsis, which creates a potential conflict between the text’s perceived distance and the screen’s physical distance, imposing a physiological load on the eye’s accommodative system that is poorly understood. While some studies have explored the objective accommodative response or the subjective perceptual experience separately (Kitaoka, 2016; Séverac Cauquil *et al*., 2009; Bai, Zhang and Li, 2016), the direct link between specific chromostereoscopic stimuli in dark interfaces and the resulting impact on perceptual ergonomics and user comfort has not been systematically quantified. This lack of evidence is a critical issue for designers and ergonomists tasked with creating interfaces that are both functional and visually sustainable.

Therefore, the primary objective of this study was to investigate the effect of different chromostereoscopic stimuli (coloured text stimuli presented on a black background) on the accommodative response in young, healthy adults. We aimed to: 1) Quantify the objective accommodative response to red, green, blue, and yellow text stimuli on a black background compared to a standard black-on-white baseline; and 2) Evaluate subjective perceptions of depth, reading comfort, and visual strain associated with each colour. We hypothesized a potential conflict between optical and perceptual drivers. While the optical principles of LCA suggest that anteriorly focused blue light should require less accommodation, we hypothesized that stimuli composed of shorter wavelengths (blue) might conversely elicit a greater accommodative response due to the difficulty in processing the blurred, low-luminance image or a shift towards the myopic ‘dark focus’ of accommodation. Whereas stimuli of longer wavelengths (red) would elicit a lesser accommodative response and be perceived as closer and more comfortable. Understanding these relationships is essential for developing evidence-based guidelines for user interface design that can optimize visual performance and minimize digital eye strain. Specifically, these findings have direct implications for the ergonomic design of digital interfaces, particularly in the development of ‘dark mode’ themes that are now ubiquitous across operating systems and applications. Furthermore, the principles derived from this research could inform the design of specialized displays such as automotive heads-up displays (HUDs) and augmented and virtual reality environments where colour cues are critical and be used in creating occupational guidelines to mitigate visual fatigue for screen-intensive professions.

## Methods

### Participants

A total of thirty young, healthy adults (11 males, 19 females) with a mean age of 19.83 ± 1.18 years (range: 18–35 years) were recruited via convenience sampling from the undergraduate and postgraduate student population to participate in this prospective, cross-sectional study. Participants were naive to the specific experimental hypothesis regarding chromostereopsis to prevent cognitive bias affecting the subjective or objective outcomes. The study was approved by the Institutional Ethics Committee, SRM Medical College Hospital and Research Centre, SRM Institute of Science and Technology, Kattankulathur, India [Ethics Clearance Number: SRMIEC-ST0224-932]. The study was conducted in accordance with the tenets of the Declaration of Helsinki.

Prior to the experimental task, each participant underwent a comprehensive eye examination, and basic orthoptic evaluation. The inclusion criteria were:

- **General and ocular health:** Free from any systemic or ocular pathology as verified by detailed history, slit lamp examination and direct ophthalmoscopy
- **Visual acuity:** Best-corrected visual acuity of 6/6 or better in each eye with habitual spectacle correction. Any participant with a residual refractive error >0.25 D was excluded
- **Refractive error:** Spherical refractive error within ±3.00 DS and astigmatism of –1.00 DC or less
- **Binocular vision status:** Normal binocular visual functions, specifically:
  • Stereoacuity ≤ 50 seconds of arc (Randot Stereotest)
  • Near Point of Convergence (NPC) ≤ 7 cm
  •Amplitude of accommodation (NPA) within normal range calculated as per Hofstetter’s equation
  • Accommodative response (measured via Monocular Estimation Method at 40 cm) within the range of +0.25 D to +0.75 D
- **Colour vision:** Normal colour perception assessed by Ishihara plates
- **Language proficiency:** Proficiency in English to ensure reading fluency during the task

### Stimulus

The experimental stimulus consisted of a continuous passage of English text, presenting a short story to ensure participant engagement during the reading task. The text was formatted in Arial font at an N8 point size (equivalent to 12 pt) with double spacing between lines to warrant optimal legibility and minimize crowding effects.

**Figure 2:**
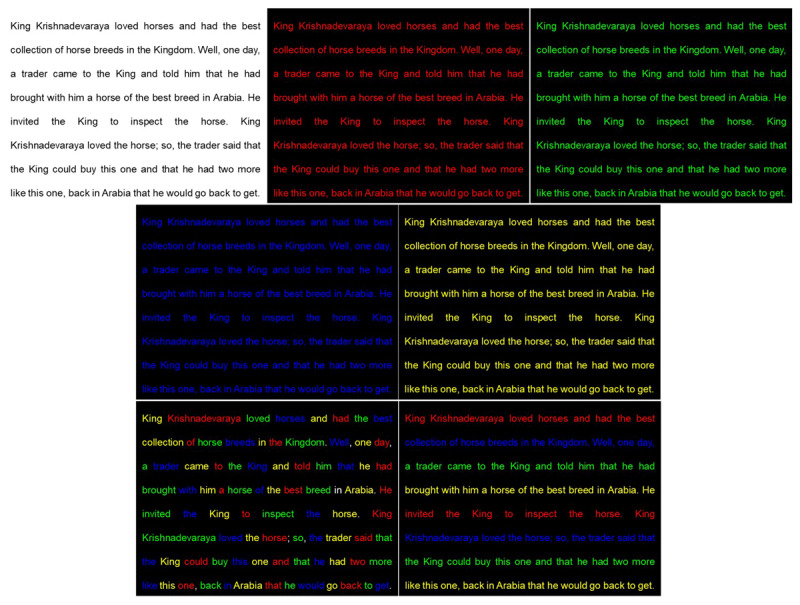
Representative screenshots of the seven stimulus conditions used in the study. From left to right: Top row: Black on White (BOW), Red on Black (ROB), Green on Black (GOB). Middle row: Blue on Black (BLOB), Yellow on Black (YOB). Bottom row: Mixed Word (BMW), Mixed Line (BSL). All text was presented in Arial font, N8 size.

As shown in Figure 2, a total of seven distinct stimulus conditions were created to evaluate the accommodative response. A standard baseline condition featured black text on a white background (BOW). The primary experimental conditions were designed to elicit a chromostereoscopic effect and consisted of coloured text on a pure black background. A black background (negative polarity) was specifically selected for these stimuli, as the perceptual depth effect of chromostereopsis is known to be most prominent and easily appreciated against a dark field when compared to a white background. This is likely because the lower overall luminance of the dark background induces pupil dilation, which, in turn, increases the magnitude of ocular aberrations and reduces the depth of focus. This renders the chromatic differences in focus more optically significant and perceptually apparent than they would be with a smaller pupil against a bright white background. The stimulus conditions used in this study are detailed in Table 1.

**Table 1:** Description of stimulus conditions and abbreviations.


STIMULUS CONDITION	ABBREVIATION	TEXT COLOUR (RGB)	BACKGROUND (RGB)

**Black on White (baseline)**	BOW	Black (0, 0, 0)	White (255, 255, 255)

**Red on Black**	ROB	Red (255, 0, 0)	Black (0, 0, 0)

**Green on Black**	GOB	Green (0, 255, 0)	Black (0, 0, 0)

**Blue on Black**	BLOB	Blue (0, 0, 255)	Black (0, 0, 0)

**Yellow on Black**	YOB	Yellow (255, 255, 0)	Black (0, 0, 0)

**Mixed Word**	BMW	Variable per word	Black (0, 0, 0)

**Mixed Line**	BSL	Variable per line	Black (0, 0, 0)


In real-world web and app design, colours are rarely luminance-matched. Hence, these non-isoluminant standard Red Green Blue (RGB) values were used to replicate the native rendering of colours in standard user interfaces on consumer devices. Two additional mixed-colour conditions were also used: one where each word was a different colour on a black background (BMW), and another where each line of text was a different colour on a black background (BSL). These conditions were designed to simulate real-world applications, such as code editors with syntax highlighting or dynamic web content, and crucially, to determine how the accommodative system responds when presented with multiple, conflicting chromostereoscopic depth cues simultaneously within the visual field.

The physical dimensions of the stimulus were precisely controlled. The height of the individual text characters was 3.3 mm, subtending a visual angle of 0.75 degrees at the specified viewing distance. The overall height of the visible text passage on the screen was 15 mm, which corresponded to a visual angle of 17 degrees.

### Experimental setup

**Figure 3:**
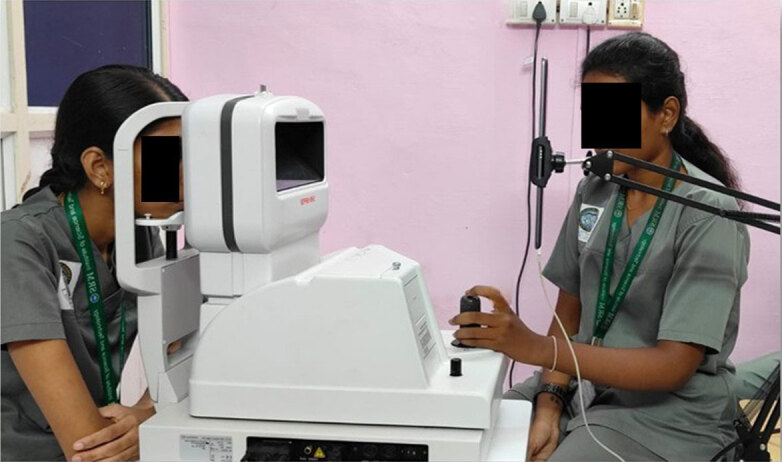
Experimental setup showing a participant viewing the iPad stimulus at a 50 cm distance through the open-field N-vision K 5001 autorefractor, with accommodation measured from the right eye.

Objective measurements of the accommodative response were obtained using an N-vision K 5001 open-field autorefractor. This instrument was chosen as it allows for objective measurement of the eye’s refractive state without obstructing the participant’s natural field of view. The experimental stimuli were presented on an Apple iPad (7^th^ generation) screen positioned on the other side of the autorefractor. Participants were seated comfortably and aligned with the instrument using an integrated chin rest and forehead rest, which ensured a stable head posture and a fixed viewing distance. The screen was precisely positioned at 50 cm from the participant’s corneal plane, creating a constant accommodative demand of 2.00 D. This distance was selected based on previous findings by Shieh and Lee (2007), who found that the preferred viewing distance for electronic displays and visual display terminals was ~50 cm. Figure 3 demonstrates the experimental setup.

While participants viewed the stimuli binocularly to simulate natural reading conditions, the accommodative response was measured exclusively from the right eye. The entire experiment was conducted under standard indoor lighting conditions, with room illumination maintained at approximately 250 lux, simulating a typical office or reading environment. Participants wore their own habitual spectacle correction throughout the experiment to ensure the stimulus was viewed with their best-corrected vision.

### Procedure

After providing written informed consent, each participant underwent the preliminary comprehensive eye examination and orthoptic evaluation to confirm their eligibility for the study. In order to measure the accommodative response, the order of presentation for the seven stimulus conditions was randomized for each participant to counteract any potential learning or fatigue effects. For each condition, the participant was seated and correctly aligned with the N-vision K 5001 autorefractor. They were instructed to read a different short story passage presented on the screen for a total duration of five minutes. Accommodative response was measured at multiple, discrete intervals throughout this period. A baseline accommodative response measurement was taken at the start of the task (0 seconds), with subsequent measurements recorded every one minute until the completion of the trial (i.e., at the 1^st^ min, 2^nd^ min, 3^rd^ min, 4^th^ min, and 5^th^ min). At each of these time points, ten consecutive measurements were taken, and the average was calculated to represent the accommodative response at that interval. To ensure active engagement and verify attention to the task, a set of three comprehension questions related to the story passage was asked immediately after each 5-minute trial. Following the completion of each five-minute trial, a minimum mandatory five-minute washout period was enforced. During this break, participants were instructed to fixate on a distant, non-accommodative target (6/60 target of the Snellen chart) to allow their accommodative system to relax and return to its baseline state before the next stimulus was presented.

Following the objective measurements and comprehension questions for a given stimulus, participants were asked to complete a subjective questionnaire. This questionnaire assessed three key perceptual factors: (1) perceived depth of the text. rated as ‘closer’ (appearing anterior to the screen plane/sticking out), ‘same’ (on the screen plane), or ‘farther’ (appearing posterior to the screen plane/depressed), (2) reading comfort, and (3) visual strain. Both comfort and strain were rated on a 5-point Likert scale, where 1 indicated ‘very uncomfortable’ or ‘very high strain’ and 5 indicated ‘very comfortable’ or ‘very low strain’. This entire sequence of reading tasks with objective measurements, comprehension questions, subjective questionnaires, and washout periods was repeated for all seven randomized stimulus conditions. The entire experimental session for each participant was completed in two visits, each lasting for approximately one hour with adequate breaks in between.

### Data Analysis

All statistical analyses were performed using the Statistical Package for the Social Sciences (SPSS) software, Version 26.0. The objective lag of accommodation data was analysed using a two-way repeated measures analysis of variance (RM-ANOVA). This test was chosen to assess the main effects of and the interaction between the two within-subjects’ factors: Stimulus Condition (with seven levels: BOW, ROB, YOB, GOB, BLOB, BMW, and BSL) and Time (with 5 levels: Baseline, 1^st^ min, 2^nd^ min, 3^rd^ min, 4^th^ min, and 5^th^ min). Mauchly’s test was used to check for violations of the assumption of sphericity, and where this assumption was not met, the Greenhouse-Geisser correction was applied to the degrees of freedom. If the RM-ANOVA revealed a significant main effect or interaction, post-hoc pairwise comparisons were conducted using a Bonferroni correction to identify specific differences between conditions while controlling for multiple comparisons. Additionally, the standard deviations of the accommodative response were considered as the variability of accommodation.

In addition to the objective measurements, subjective ratings were collected to assess participants’ perceptual experience. Participants were asked to identify which of the primary-coloured stimuli appeared ‘closest’, ‘farthest’, ‘most easy to read’, and ‘most difficult to read’. The responses were analysed, and the percentage of participants selecting each colour for each category was calculated. The alpha level for determining statistical significance was set at p < 0.05 for all tests.

## Results

A total of 30 participants completed the study, and data from all participants were included in the final analysis. The primary outcome measure was the lag of accommodation, calculated as the difference between the stimulus demand (2.00 D) and the objectively measured accommodative response. A larger lag value indicates a greater degree of under-accommodation and poorer focusing accuracy.

### Objective accommodative response

A seven (stimulus condition) × six (time: 0, 1, 2, 3, 4, 5 minutes) RM-ANOVA was conducted to analyse the accommodative response. Mauchly’s test of sphericity indicated that the assumption of sphericity had been violated for the main effect of stimulus (χ² (20) = 32.20, p = 0.04), the main effect of time (χ² (14) = 28.46, p = 0.01), and the interaction between stimulus and time (p < .001). Therefore, all reported F-statistics are based on degrees of freedom, corrected using the Greenhouse-Geisser correction.

#### Main effect of stimulus condition

The analysis revealed a statistically significant and strong main effect of stimulus condition on the lag of accommodation (F (4.36, 126.35) = 17.43, p < .001, partial η² = 0.38). This indicates that the choice of text and background colour combination had a significant impact on the participants’ mean accommodative state across the reading task.

Post-hoc pairwise comparisons with Bonferroni correction were used to examine the differences between specific stimuli. The estimated marginal means from the analysis showed that the ROB condition produced the smallest mean accommodative lag (0.18 D), while the BLOB condition produced the greatest mean accommodative lag (0.61 D).

Table 2 shows the mean accommodative lag for all 7 stimulus conditions. The baseline BOW condition resulted in an intermediated mean lag of 0.35 D. The following key significant differences were observed:

**Table 2:** Mean accommodative lag and standard deviation for each stimulus condition.


STIMULUS CONDITION	ACCOMMODATIVE LAG (D)	STANDARD DEVIATION (D)

**Red on Black (ROB)**	0.18	0.51

**Black on White (BOW)**	0.35	0.56

**Mixed Line (BSL)**	0.35	0.54

**Mixed Word (BMW)**	0.35	0.56

**Yellow on Black (YOB)**	0.36	0.55

**Green on Black (GOB)**	0.44	0.58

**Blue on Black (BLOB)**	0.61	0.60

- The BLOB condition elicited a significantly greater accommodative lag (0.61 D) compared to BOW (0.35 D, p < .001), BSL (0.35 D, p < .001), BMW (0.35 D, p < .001), ROB (0.18 D, p < .001), YOB (0.36 D, p < .001), and GOB (0.44 D, p = .003).
- Conversely, the ROB condition resulted in a significantly smaller accommodative lag (0.18 D) compared to the other coloured text stimuli: BSL (0.35 D, p = .025), BMW (0.35 D, p = .038), YOB (0.36 D, p = .007), GOB (0.44 D, p < .001), and BLOB (0.61 D, p < .001). The lag for ROB was not significantly different from the baseline BOW condition (0.35 D, p = .119).
- The GOB condition (0.44 D) induced a significantly greater lag than ROB (0.18 D, p < .001) and a significantly smaller lag than BLOB (0.61 D, p = .003), positioning it between the two spectral extremes.
- The accommodative lags induced by the BOW (0.35 D), BSL (0.35 D), and BMW (0.35 D), and YOB stimulus (0.36 D) were not statistically different from one another (p > .99 for all pairwise comparisons).

#### Main effect of time

There was no significant main effect of time on the accommodative lag (F (3.39, 98.24) = 0.61, p = 0.63, partial η² = 0.02). This suggests that, when averaged across all stimulus conditions, the accommodative lag did not significantly change over the five-minute reading period.

#### Main effect of stimulus and time interaction

**Figure 4:**
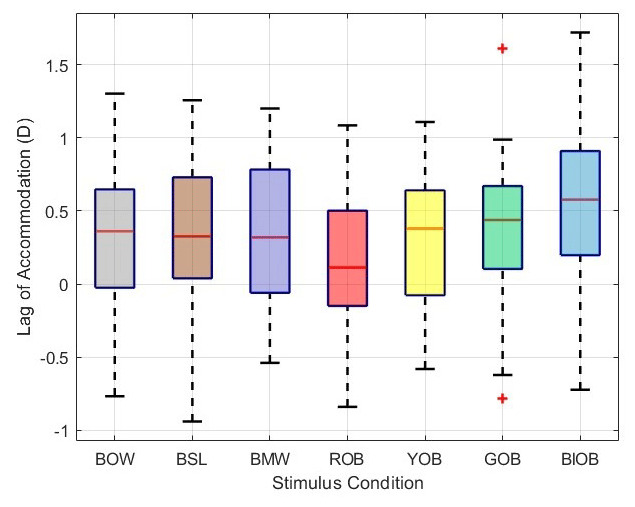
Estimated marginal means of accommodative response for each stimulus condition measured over the five-minute task. The y-axis represents the mean accommodative lag in dioptres. (BOW: Black on White, ROB: Red on Black, YOB: Yellow on Black, GOB: Green on Black, BLOB: Blue on Black, BMW: Each word was a different colour on a black background and in BSL: Each line of text was a different colour on a black background).

Furthermore, the stimulus-time interaction was not statistically significant after applying the Greenhouse-Geisser correction (F (11.45, 332.15) = 1.52, p = .12, partial η² = .05). Although the mean accommodative lag varied between stimuli, this pattern of differences remained consistent across the 5-minute measurement period, as illustrated in the profile plot (Figure 4).

### Accommodation variability

The ANOVA revealed that there was no statistically significant effect of the stimulus condition on the variability of accommodation, F (3.30, 16.50) = 1.86, p = 0.17, with a partial eta squared of 0.27.

Although the main effect was not significant, descriptive statistics (Table 2) showed a trend where the BLOB condition elicited the highest mean variability (SD = 0.60 D) and the ROB condition elicited the lowest (SD = 0.51 D). However, subsequent pairwise comparisons with Bonferroni correction confirmed the main ANOVA result, revealing no significant differences in accommodative variability between any of the stimulus conditions (p > .05 for all comparisons). This suggests that while different coloured stimuli significantly impacted the accuracy (lag) of accommodation, they did not significantly alter the stability (variability) of the response in this study.

### Subjective preference of perceived depth and reading difficulty

**Figure 5:**
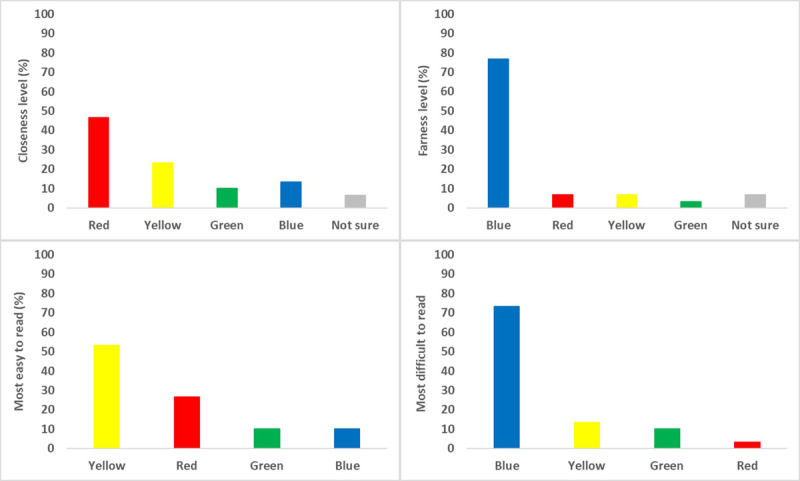
Percentage of participants selecting each colour stimulus for perceived closeness (top left), farness (top right), being the easiest to read (bottom left), and being the most difficult to read (bottom right).

In addition to the objective measurements, participants provided subjective ratings regarding the perceived depth and ease of reading for each of the primary-coloured stimuli (Figure 5).

### Perceived depth

Participant ratings of perceived depth strongly aligned with the established principles of chromostereopsis. When asked which colour appeared ‘closest’, red was the most frequently selected stimulus, chosen by approximately 46.67% of participants. Yellow was the second most-cited colour for ‘closeness’ (23.33%). Conversely, when asked which colour appeared ‘farthest’, blue was the overwhelming choice, selected by approximately 76.67% of participants. These clear and opposing perceptions for red and blue stimuli provide strong subjective evidence for the chromostereoscopic effect under the experimental conditions.

### Reading ease and difficulty

The subjective ratings for reading comfort also revealed a distinct pattern of preference. Yellow was most frequently identified as the ‘most easy to read’ colour, selected by a clear majority of participants (53.33%). Red was the second most-preferred colour for reading ease (26.67%). In contrast, blue was overwhelmingly rated as the ‘most difficult to read’, with approximately 73.33% of participants choosing it for this category.

Overall, the subjective data show a strong relationship between colour, perceived depth, and reading comfort. The blue stimulus, which was perceived as farthest away, was also rated as the most difficult to read, while the yellow and red stimuli were perceived as easier to read.

## Discussion

This study aimed to investigate the effect of different coloured text stimuli presented on a black background on the accommodative response and subjective visual perception in young, healthy adults. Our results demonstrate a profound and systematic influence of stimulus colour on the accuracy of accommodation. The primary finding was that against the black background the short-wavelength blue stimulus induced the greatest accommodative lag, while the long-wavelength red stimulus induced the smallest. These objective findings were strongly corroborated by subjective reports, where blue was perceived as the most difficult to read and the most distant.

The most striking result of this study is the large accommodative lag observed for the BLOB condition. The principle of LCA states that since short-wavelength blue light focuses anterior to the retina, the eye’s optical system should relax accommodation to shift the focal point back onto the retinal plane (Maier *et al*., 2015). Our findings are consistent with this optical prediction, as the accommodative system exerted the least effort, resulting in a significant under-accommodation (lag) of over 0.60 D.

From a clinical perspective, however, this magnitude of lag is significant. It exceeds the typical physiological depth of focus (approximately ±0.50D), implying that the retinal image is not merely relaxed but perceptibly blurred. This suggests that for an isolated, monochromatic stimulus on a dark background, the accommodative system may not be responding to LCA in its canonical manner. One potential explanation is that pure blue text, having lower effective luminance and being at the edge of the visible spectrum, acts as a poor or inadequate stimulus for the accommodative feedback loop (Shapley *et al*., 2024; Simonet and Campbell, 1990). Without a clear, high-contrast signal to drive focus, the system may revert to its resting or tonic state of accommodation, leading to a large accommodative lag.

A second, complementary explanation involves the powerful perceptual cue of chromostereopsis. Our subjective data confirmed that participants overwhelmingly perceived the blue text as being farther away. This strong, top-down perceptual signal of distance may have overridden the bottom-up physiological need to accommodate for a near target. The conflict between the screen’s physical distance and the colour’s perceived distance could lead the visual system to adopt a more relaxed accommodative posture, further contributing to the large measured lag. This stands in contrast to Jiménez *et al*. (2020), who found a heightened accommodative response for blue-red colour combinations. The critical difference may be that their stimuli involved chromatic contrast (e.g., blue text on a red background), which provides a robust signal for the visual system to act upon, whereas our study used isolated colours against a neutral black background, a condition increasingly common in dark mode interfaces.

In contrast, the results for the ROB stimulus align well with LCA theory. This mechanism parallels the principles of the clinical duochrome test, where the longer wavelength (red) focuses effectively on the retina (or slightly behind it). Specifically, red light focuses slightly behind the retina (hyperopic defocus). To achieve clarity, the accommodative system must increase its effort to pull the focal point forward onto the retina. This increased accommodative demand was reflected in our data as the smallest accommodative lag (0.18 D), indicating the most accurate focusing performance among all conditions. The fact that the lag for ROB was not significantly different from the standard BOW baseline suggests that it is a highly effective stimulus for accommodation.

Similarly, the YOB, GOB, and mixed-colour (BSL, BMW) stimuli all produced accommodative lags that were statistically indistinguishable from the BOW baseline. This finding for yellow supports long-standing recommendations in low vision rehabilitation, where yellow text on dark backgrounds is prescribed due to its high luminance contrast. The yellow stimulus lies close to the peak of the photopic luminosity function. Furthermore, unlike the single-channel red or blue stimuli, the yellow stimulus is generated by activating both red and green sub-pixels on the display. This additive mixing results in a higher luminance target, providing a high-contrast luminance signal that effectively drives the accommodative system, unlike the low-luminance blue stimulus.

The synergy between our objective and subjective findings is a major strength of this study. The colour that produced the greatest physiological deficit (the largest accommodative lag for blue) was unequivocally rated as the most difficult to read and perceived as the most distant. This provides a direct link between accommodative inaccuracy and the subjective experience of visual difficulty. The brain’s struggle to achieve a clear focus on the blue target manifests perceptually as discomfort and poor legibility. Conversely, yellow, which produced a ‘normal’ accommodative lag similar to the baseline, was rated as the easiest to read, reinforcing the idea that effective accommodation is a prerequisite for comfortable vision.

Several limitations of this study must be acknowledged. First, the stimuli were presented on a commercial tablet display (Apple iPad) rather than a calibrated psychophysical monitor. While this was done to maximize the ecological validity of the study and simulate the real-world conditions under which digital eye strain occurs, it introduces optical limitations. We used standard RGB values to define our stimuli; we did not perform spectroradiometric measurements of the precise luminance and chromaticity of each colour on the iPad display. Such data would strengthen the study’s reproducibility and allow for more direct comparisons with other research. Second, our sample consisted of young, healthy university students. These findings may not be generalizable to older populations with presbyopia or individuals with pre-existing accommodative or binocular vision disorders. Finally, while our 5-minute reading task was longer than that of some previous studies, it may not be sufficient to induce significant, measurable visual fatigue. Future research should explore these effects over longer durations, and it should also explore the effects of positive polarity for a comprehensive comparison.

Despite these limitations, this study has direct and significant implications for the ergonomic design of digital interfaces. Our findings strongly recommend avoiding the use of pure, saturated blue text, particularly for fine details or extended reading on dark backgrounds. It places a significant and unsupported demand on the accommodative system, leading to poor focusing accuracy and subjective discomfort. In contrast, colours like red and yellow appear to be more effective and comfortable choices for text in dark mode environments. These evidence-based insights can help UI/UX designers, software developers, and content creators develop visual displays that are not only aesthetically pleasing but are also physiologically optimized for visual performance and comfort. Future studies should build upon these findings by investigating the interaction between luminance and colour on accommodative response, exploring a wider range of background colours, and examining the concurrent response of the vergence system.

## Conclusion

This study demonstrates that colour is not merely a stylistic choice in digital design but a critical factor in the visual ergonomics of digital interfaces, significantly influencing the physiological function of the human eye. The choice of coloured text on a dark background directly impacts accommodative response in a systematic, wavelength-dependent manner. Specifically, we found that short-wavelength (blue) text induced a significant accommodative lag, while long-wavelength (red) text prompted the most accurate accommodative response. Interestingly, while red text was optically superior, yellow text was subjectively rated as the easiest to read, likely due to its higher luminance contrast.

This objective deficit was strongly mirrored in subjective perceptions, with blue text being rated as the most difficult to read and perceived as most distant. Therefore, the principal conclusion of this work is that while chromostereopsis provides a compelling perceptual experience, the use of pure, short-wavelength text on dark backgrounds acts as a poor stimulus for accommodation, leading to significant focusing errors and a diminished user experience.

## Declaration of Generative AI and AI-Assisted Technologies in the Writing Process

During the preparation of this work, the authors used ChatGPT and Google AI Studio in order to improve the readability and language of the manuscript. After using this tool/service, the authors reviewed and edited the content as needed and take full responsibility for the content of the published article.

## Data Accessibility Statement

All data related to this study are considered confidential and will be made available upon reasonable request from the corresponding author.

## References

[1] Allen, R.C. and Rubin, M.L. (1981) ‘Chromostereopsis’, Survey of Ophthalmology, 26, pp. 22–27. Available at: 10.1016/0039-6257(81)90121-1 7280992

[2] Bai, Y., Zhang, Y. and Li, Z. (2016) ‘Perceived depth modeling based on chromostereopsis’, 2016 1nd IEEE International Conference on Computer and Communications (ICCC). Chengdu, China, 14–17 October. Piscataway, NJ: Institute of Elecrical and Electronics Engineers, pp. 723–727. Available at: 10.1109/CompComm.2016.7924797

[3] Damnjanović, Đ., Stojić, D., Vujičić, D. and Milošević, M. (2024) ‘Minimalistic user interface design and dark mode usage in human-computer interaction’, 10th International Scientific Conference Technics, Informatic, and Education. Presented at the Proceedings TIE 2024. Čačak, Serbia, 20–22 September. Kragujevac, Serbia: University of Kragujevac, pp. 124–128. Available at: 10.46793/TIE24.124D

[4] Dobres, J., Chahine, N. and Reimer, B. (2017) ‘Effects of ambient illumination, contrast polarity, and letter size on text legibility under glance-like reading’, Applied Ergonomics, 60, pp. 68–73. Available at: 10.1016/j.apergo.2016.11.001 28166901

[5] Howarth, P.A. and Bradley, A. (1986) ‘The longitudinal chromatic aberration of the human eye, and its correction’, Vision Research, 26(2), pp. 361–366. Available at: 10.1016/0042-6989(86)90034-9 3716229

[6] Hynes, N.J., Cufflin, M.P., Hampson, K.M. and Mallen, E.A. (2022) ‘The effect of image resolution of display types on accommodative microfluctuations’, Ophthalmic Physiological Optics, 42(3), pp. 514–525. Available at: 10.1111/opo.12949 35107178 PMC9302673

[7] Jiménez, R., Redondo, B., Molina, R., Martínez-Domingo, M.Á., Hernández-Andrés, J. and Vera, J. (2020) ‘Short-term effects of text-background color combinations on the dynamics of the accommodative response’, Vision Research, 166, pp. 33–42. Available at: 10.1016/j.visres.2019.11.006 31841707

[8] Kahal, F., Al Darra, A. and Torbey, A. (2025) ‘Computer vision syndrome: A comprehensive literature review’, Future Science OA, 11(1), 2476923. Available at: 10.1080/20565623.2025.2476923 40055942 PMC11901492

[9] Karwoski, T.F. and Lloyd, V.V. (1951) ‘Studies in vision: V. The role of chromatic aberration in depth perception’, The Journal of General Psychology, 44(2), pp. 159–173. Available at: 10.1080/00221309.1951.9710067

[10] Kitaoka, A. (2016) ‘Chromostereopsis’, in M.R. Luo (ed.) Encyclopedia of color science and technology. New York, NY: Springer, pp. 114–125. Available at: 10.1007/978-1-4419-8071-7_210

[11] Labhishetty, V., Cholewiak, S.A., Roorda, A. and Banks, M.S. (2021) ‘Lags and leads of accommodation in humans: Fact or fiction?’ Journal of Vision, 21, pp. 1–18. Available at: 10.1167/jov.21.3.21 PMC7995353 33764384

[12] Landrum, B.T. (2009) The effect of letter size on the accommodative response. Master’s thesis. Ohio State University. Available at: http://rave.ohiolink.edu/etdc/view?acc_num=osu1243026823

[13] Maier, F.M., Howland, H.C., Ohlendorf, A., Wahl, S. and Schaeffel, F. (2015) ‘Lack of oblique astigmatism in the chicken eye’, Vision Research, 109(A), pp. 68–76. Available at: 10.1016/j.visres.2015.02.002 25701740

[14] Malter, A.J. and Rindfleisch, A. (2019) ’Transitioning to a digital world’, in A. Rindfleisch and A.J. Malter (eds.) Review of marketing research. Bingley, UK: Emerald Publishing Limited, pp. 1–11. Available at: 10.1108/S1548-643520190000016001

[15] Molina, P.B., Taboada, J.J.E., Blasco, T.F. and Mico, R.M. (2019) ‘Influence of contrast polarity on accommodative response’, Journal of Optometry, 12(1), pp. 38–43. Available at: 10.1016/j.optom.2018.03.002 29627300 PMC6318541

[16] Muhamad, N. and Mokhtar, N. (2024) ‘The effect of display polarity on reading speed and reading error among young adults’, Journal of Health Science and Medical Research, 42(6), 20241095. Available at: 10.31584/jhsmr.20241095

[17] Muhamad, N., Moktaeffendi, N.H. and Azni, N.S. (2023) ‘Effect of display polarity on amplitude of accommodation and visual fatigue’, in Environment-Behaviour Proceedings Journal. Presented at the 11th AMER International Conference on Quality of Life (AicQoL2023). Bangkok, Thailand, 28–30 April. UK: e-International Publishing, pp. 207–214. Available at: 10.21834/ebpj.v8i24.4611

[18] Seidemann, A. and Schaeffel, F. (2002) ‘Effects of longitudinal chromatic aberration on accommodation and emmetropization’, Vision Research, 42(21), pp. 2409–2417. Available at: 10.1016/S0042-6989(02)00262-6 12367740

[19] Séverac Cauquil, A., Delaux, S., Lestringant, R., Taylor, M.J. and Trotter, Y. (2009) ‘Neural correlates of chromostereopsis: An evoked potential study’, Neuropsychologia, 47(12), pp. 2677–2681. Available at: 10.1016/j.neuropsychologia.2009.05.002 19442677

[20] Shapley, R., Nunez, V. and Gordon, J. (2024) ‘Low luminance contrast’s effect on the color appearance of S-cone patterns’, Vision Research, 222, p. 108448. Available at: 10.1016/j.visres.2024.108448 38906035

[21] Shieh, K.-K. and Lee, D.-S. (2007) ‘Preferred viewing distance and screen angle of electronic paper displays’, Applied Ergonomics, 38(5), pp. 601–608. Available at: 10.1016/j.apergo.2006.06.008 17049333

[22] Simonet, P. and Campbell, M.C.W. (1990) ‘Effect of illuminance on the directions of chromostereopsis and transverse chromatic aberration observed with natural pupils’, Ophthalmic and Physiological Optics, 10(3), pp. 271–279. Available at: 10.1111/j.1475-1313.1990.tb00863.x 2216476

[23] Summersgill, K.L. and Keating, P.D. (2011) ‘Chromostereopsis and stereograms’, British and Irish Orthoptic Journal, 8, pp. 50–53. Available at: 10.22599/bioj.41

[24] Thibos, L.N., Bradley, A., Still, D.L., Zhang, X. and Howarth, P.A. (1990) ‘Theory and measurement of ocular chromatic aberration’, Vision Research, 30(1), pp. 33–49. Available at: 10.1016/0042-6989(90)90126-6 2321365

[25] Thompson, P., May, K. and Stone, R. (1993) ‘Chromostereopsis: A multicomponent depth effect?’ Displays, 14(4), pp. 227–234. Available at: 10.1016/0141-9382(93)90093-K

[26] Wagner, S., Schaeffel, F., Zrenner, E. and Straßer, T. (2019) ‘Prolonged nearwork affects the ciliary muscle morphology’, Experimental Eye Research, 186, p. 107741. Available at: 10.1016/j.exer.2019.107741 31336108

[27] Wajuihian, S.O. and Hansraj, R. (2015) ‘A review of non-strabismic accommodative-vergence anomalies in school-age children. Part 1: Vergence anomalies’, African Vision and Eye Health, 74(1), p. a32. Available at: 10.4102/aveh.v74i1.32

[28] Westermann, H. (2022) Using chromostereopsis to enhance depth perception in photos by changing the hue. PhD dissertation. Delft University of Technology.

[29] Zaman, M.N., Kalicheti, S. and Goyal, A. (2025) ‘Impact of screen brightness on static and dynamic aspects of accommodation among digital eye strain subjects’, Communications on Applied Nonlinear Analysis, 32, pp. 728–739. Available at: 10.52783/cana.v32.5100

[30] Zhou, Y., Zhang, J. and Fang, F. (2021) ‘Vergence-accommodation conflict in optical see-through display: Review and prospect’, Results in Optics, 5, p. 100160. Available at: 10.1016/j.rio.2021.100160

